# Altered Functional Connectivity in Pain-Related Brain Regions and Its Correlation with Pain Duration in Bone Metastasis with Cancer Pain

**DOI:** 10.1155/2022/3044186

**Published:** 2022-08-28

**Authors:** Xiaoyu Zhou, Yong Tan, Jiao Chen, Chengfang Wang, Yu Tang, Jiang Liu, Xiaosong Lan, Hong Yu, Yong Lai, Yixin Hu, Jing Zhang, Ying Cao, Daihong Liu, Jiuquan Zhang

**Affiliations:** Department of Radiology, Chongqing University Cancer Hospital, School of Medicine, Chongqing University, Chongqing 400030, China

## Abstract

Bone metastatic pain is thought to be a severe type of cancer pain that has refractory characteristics and a long duration. This study is aimed at exploring the brain functional connectivity (FC) pattern in lung cancer patients with bone metastatic pain. In this study, 27 lung cancer patients with bone metastatic pain (CP+), 27 matched lung cancer patients without pain-related complaints (CP−), and 27 matched healthy controls (HC) were recruited. All participants underwent fMRI data acquisition and clinical assessments. One-way ANOVA or a Mann–Whitney *U* test was applied to compare clinical data according to data distribution. Seventeen hypothesis-driven pain-related brain regions were selected as regions of interest (ROIs). FC values among pain-related brain regions across the three groups were computed by using ROI–ROI functional connectivity analysis. ANCOVA with a post hoc test was applied to compare FC differences among the three groups. *p* < 0.05 indicated statistical significance. Correlation analysis was conducted to explore the potential relationship between the FC values and clinical characteristics. Except for years of education, no significant differences were revealed among the three groups in age, gender, or neuropsychological assessment. In the CP+ group, FC alterations were mainly concentrated in the dorsal lateral prefrontal cortex (DLPFC), anterior cingulate cortex (ACC), secondary somatosensory cortex (SII), and amygdala compared to the CP− group. Among these brain regions with statistical differences, FC between the right DLPFC and the right ACC showed a positive correlation with the duration of cancer pain in the CP+ group. In addition, in the CP− group, altered FC was found in the bilateral SII, ACC, and thalamus compared to the HC group. Altered FC in pain-related brain regions may be a brain pattern of bone metastatic pain and may be associated with the long duration of cancer pain.

## 1. Introduction

Pain is a common symptom in cancer patients. Approximately 64% of cancer patients with advanced cancer or metastases report cancer pain [1]. Bone metastatic pain is thought to be a severe type of cancer pain that has refractory characteristics with a long duration [2]. Therefore, exploring the mechanism of bone metastatic pain is of great significance for early prevention and intervention.

The pathophysiological mechanisms of bone metastatic pain have begun to be elucidated. Nociceptors are activated by bone metastasis itself or secreted pain-inducing mediators [3]. In addition, acid-sensitive ion channels of nociceptors are activated by acids produced during bone remodeling [4]. Beyond that, peripheral and central sensitization increases the sensitivity of pain [5]. The above pathological abnormalities eventually manifest as pain perceptions and unpleasant emotions, which can be generated and regulated by the brain. The function and structure of the brain change can accordingly be detected by magnetic resonance imaging (MRI).

Neuroimaging studies of patients with pain have suggested that pain is closely related to alterations in brain function and structure [6, 7]. Brain regions processing pain-related information were considered as a “pain matrix” as follows. The thalamus, posterior insula, and primary somatosensory cortex (SI) can receive nociceptive afferent information and then encode pain intensity. The anterior cingulate gyrus (ACC), anterior insula, and secondary somatosensory cortex (SII) can manage unpleasant pain-related emotions. Midbrain periaqueductal gray matter (PAG) is an important hub for pain regulation [8]. Moreover, the amygdala and prefrontal cortex participate in processing pain-related emotion, memory, and fear [9]. A large number of studies have explored the abnormal brain function within the pain matrix in various types of chronic pain. In general, stronger functional connectivity of the pain-related brain areas was demonstrated in patients with pain compared to controls in most instances [10–14]. Functional connectivity manifests differently in different pain conditions and has a potential to assist in diagnostic classification [15]. However, neuroimaging studies on pain have mainly focused on noncancer chronic pain and have paid little attention to cancer pain.

The prevalence of chronic pain among adult cancer survivors is nearly double that of adults without a cancer diagnosis [16]. Previous studies have explored the cerebral structure [17] and functional connectivity [18] of chronic neuropathic pain after surgery in breast cancer patients with or without psychological interventions. The above studies found that higher fractional anisotropy (FA) values in the left subcortical regions [17] and greater posterior cingulate connectivity with medial prefrontal regions [18] are both associated with a reduction in pain perception after psychological interventions. Previous studies on brain function in bone metastatic pain have been carried out using animal models. In studies of a mouse model with chronic pain from bone metastasis, abnormalities in functional connectivity were found in the PAG, amygdala, thalamus, and somatosensory cortex [19]. In mice with bone metastatic pain, the prefrontal cortex, cingulate cortex, and ventral striatum are regarded as central regions participating in pain-related network remodeling [20]. To our knowledge, functional connectivity abnormalities in patients with bone metastatic pain have not yet been reported.

Due to the long duration and the special pathophysiological mechanisms of bone metastatic pain, it is necessary to explore its neuroimaging mechanism. As far as we know, functional connectivity analysis has been reported in a mouse model of metastatic bone cancer, however, not in the human population. This study provides a first attempt to explore the functional connectivity within pain-related brain regions in lung cancer patients with bone metastasis suffering from cancer pain by using region of interest- (ROI-) ROI functional connectivity analyses. We hypothesized that abnormalities in functional connectivity within pain-related brain regions may occur in lung cancer patients with bone metastatic pain and that altered functional connectivity may correlate with pain-related clinical characteristics.

## 2. Methods

### 2.1. Participants

Participants were lung cancer patients with bone metastases suffering from cancer pain (CP+), lung cancer patients without pain-related complaints (CP−), and healthy controls (HC). The above three groups were matched by age and gender. All participants were recruited at Chongqing University Cancer Hospital from August 2020 to January 2022. All patients (both CP+ and CP−) were pathologically confirmed to have lung cancer. On the basis of pathologically diagnosed lung cancer, patients with bone metastasis were diagnosed when meeting one of two conditions: (a) the results of bone lesion biopsy showed lung cancer metastasis and (b) typical imaging manifestations of bone metastasis. Pain perception was assessed by the Numeric Rating Scale (NRS) for all participants. The CP+ group should meet NRS ≥ 1, while the CP− and HC group need to meet NRS = 0. All CP+ patients received regular analgesic therapy. Exclusion criteria were as follows: (a) intracranial metastases, encephalatrophy, trauma, or a history of brain surgery; (b) a history of psychiatric disorders; (c) extensive head motion (a maximum rotation greater than 3° or a maximum displacement greater than 3 mm); and (d) claustrophobia. This study was approved by the Research Ethical Committee of Chongqing University Cancer Hospital (IRB: CZLS2021042). Signed informed consent was obtained from all participants.

Of the 40 lung cancer patients with bone metastatic pain who were initially recruited for this study, seven patients had brain metastasis, four patients were diagnosed with encephalatrophy, and two patients had head motion displacement greater than 3 mm. Of the 35 recruited lung cancer patients without cancer pain, four patients had brain metastases, one patient had a history of cerebral hemorrhage, and three patients had head motion displacement greater than 3 mm. Of the recruited healthy volunteers, 27 matched volunteers were selected for this study. Therefore, 27 lung cancer patients with bone metastatic pain, 27 lung cancer patients without cancer pain, and 27 healthy volunteers were included in this study.

### 2.2. Clinical Assessments

Clinical pain, depression, and anxiety assessments and MRI scans for each participant were acquired on the same day. NRS was adopted to evaluate pain intensity. The NRS includes 11 numbers, and 0 represents “no pain” to 10 represents “the worst pain imaginable.” The participants scored the pain intensity under the guidance of an experienced physician. The duration of cancer pain in the CP+ group was calculated according to the medical record. Anxiety and depression statuses were assessed by the Self-Rating Anxiety Scale (SAS) and Self-Rating Depression Scale (SDS), respectively. Demographic information of all participants was obtained by the self-report questionnaire.

### 2.3. Resting-State Functional MR Data Acquisition

Acquisition MRI scans were examined on a 3.0 T scanner (Magnetom Prisma; Siemens Healthcare, Erlangen, Germany) equipped with a 64-channel head-neck coil. Participants were instructed to remain motionless, close their eyes, stay awake, and avoid thinking about any topics. Earplugs were used to alleviate the influence of noise, and cushions were used to restrict head motion. Three sequences were collected as follows. First, the structural information was acquired to guide subsequent functional imaging with a T1-weighted three-dimensional magnetization prepared rapid gradient echo (MPRAGE) sequence: repetition time (TR) = 2100 milliseconds (ms), echo time (TE) = 2.26 ms, flip angle = 8°, field of view (FOV) = 256 × 256 mm^2^, matrix = 256 × 256, slice thickness = 1 mm with no slice gap, and slices = 192. The total scanning time was 4 minutes and 53 seconds. Second, rapid gradient echo-planar pulse imaging (EPI) was used to acquire the resting-state blood oxygenation level-dependent (BOLD) signal: TR = 2000 ms, TE = 30 ms, flip angle = 70°, FOV = 240 × 240 mm^2^, slices = 36 (interleaved), matrix = 80 × 80, and voxel size = 3 × 3 × 3 mm^3^. From each participant, 240 volumes were acquired over 8 minutes and 8 seconds. Additionally, routine axial T2-weighted images were obtained to exclude subjects with intracranial metastases or other lesions, as demonstrated by the exclusion criteria above.

### 2.4. Brain Region Masks

**Figure 1:**
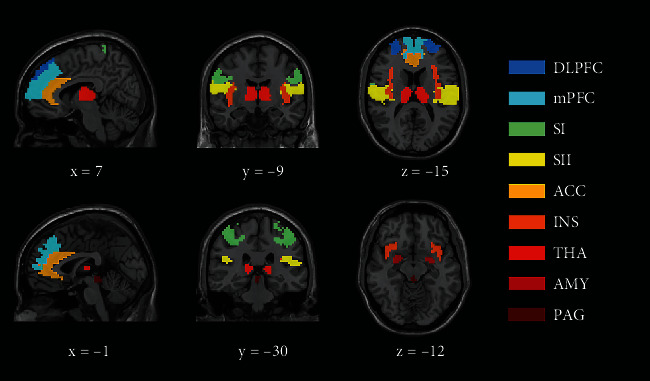
The pain-related brain region masks. DLPFC: dorsal lateral prefrontal cortex; mPFC: medial prefrontal cortex; SI: primary somatosensory cortex; SII; secondary somatosensory cortex; ACC: anterior cingulate cortex; INS: insula; THA: thalamus; AMY: amygdala; PAG: periaqueductal gray.

Based on functional connectivity analysis results from previous pain-related studies, 17 hypothesis-driven pain-related brain regions were selected as ROIs in the current fMRI study. The selected brain regions included the bilateral thalamus, bilateral insula, bilateral amygdala, bilateral ACC, bilateral SI, bilateral SII, bilateral dorsal lateral prefrontal cortex (DLPFC), bilateral medial prefrontal cortex (mPFC), and PAG. Detailed information on these pain-related brain regions is shown in Table 1. Masks for most pain-related regions were selected from the Anatomical Automatic Labeling (AAL) Atlas, including the bilateral thalamus, insula, amygdala, and ACC. In addition, the masks for bilateral SI and SII were chosen from the Juelich Histological Atlas [21], which was distributed with the FMRIB Software Library (FSL) tool. In addition, the bilateral DLPFC and mPFC were selected from the Brainnetome Atlas [22]. Finally, the PAG mask was chosen from the Harvard Ascending Arousal Network Atlas [23]. The selected brain region masks are shown in Figure 1.

### 2.5. Processing of Resting-State fMRI Data

The preprocessing of the BOLD data was carried out using the Data Processing Assistant for Resting-State fMRI (DPARSF, http://rfmri.org/DPARSF) based on MATLAB R2020a. All process followed a standard procedure as described in previous studies [24]. Briefly, we first converted all DICOM files into NifTI files. Then, the first 10 volumes of each participant were removed to reach signal equilibration. A correction was made for time differences between slices, and then, the scans were realigned to the middle point to account for head motion. The head motion parameters of all participants were then assessed, and participants with a maximum rotation greater than 3° or a maximum displacement greater than 3 mm were excluded. The head motion parameter, mean framewise displacement by Jenkinson, of the remaining subjects was extracted for the subsequent step. We performed nuisance regression separately on white matter and cerebrospinal fluid. The motion-corrected BOLD images were spatially normalized and then resampled with a voxel size of 3 × 3 × 3 mm^3^. To reduce spatial noise, the images were spatially smoothed with a Gaussian kernel of 6 mm full width at half maximum (FWHM). A bandpass filter was set as 0.01 Hz < *f* < 0.10 Hz to remove the influence of low-frequency physiological drift and high-frequency noise. After these steps, a 4-dimensional residual time series dataset was created in the standard MNI space. Intragroup functional connectivity ROI-ROI analysis was conducted among the 17 pain-related brain regions using RESTplus V1.22 (http://www.restfmri.net). For each ROI, the mean time series was calculated and then correlated with the other ROIs for each subject. By using Fisher's *r*-to-*z* transformation, Pearson correlation coefficients were converted to normally distributed scores.

### 2.6. Statistical Analysis

All data were analyzed using the statistical program SPSS 25.0. For intergroup comparisons of demographic data and neuropsychological test scores, the Shapiro–Wilk test was used to verify the normality of the data. Subject characteristics were compared among three groups using ANOVA or the Mann–Whitney *U* test depending on their distributions. The gender proportion was examined using the chi–square test. *p* < 0.05 indicated statistical significance.

For intergroup comparisons of functional connectivity, the normally distributed *z* scores of functional connectivity between two ROIs were analyzed using one-way ANCOVA among the three groups. Age [25, 26] and gender [24, 27] were regarded as important factors in pain perception. Education in years was different among three groups in this study. And diversity in fMRI results was thought to covary with subject movements [28]. Because of the above reasons, age, gender, education in years, and head motion parameters were set as covariates. The post hoc LSD test was used to identify the relationship between each group pairing. The Kruskal–Wallis ANCOVA (with covariates controlled) and a post hoc Manne–Whitney *U* tests were used to analyze *z* scores with nonnormal distributions. The above ANCOVA tests were also controlled for multiple comparisons with a Bonferroni correction of *p* < 0.003 (resulting from *p* = 0.05/17 functional connectivity measures).

A partial correlation was conducted on the CP+ group to evaluate the association between the transformed *z* scores of functional connectivity and clinical characteristics (pain intensity, duration of cancer pain, and scores on the SAS and SDS). Age, gender, education in years, and head motion parameters were set as covariates. *p* < 0.05 was considered as statistically significant.

## 3. Results

### 3.1. Demographic Data

The demographic and clinical characteristics of the participants are detailed in Table 2. No significant differences were revealed among the three groups in regard to age, gender, or neuropsychological assessment. Years of education among the three groups showed statistical significance.

### 3.2. Functional Connectivity in the Three Groups

**Figure 2:**
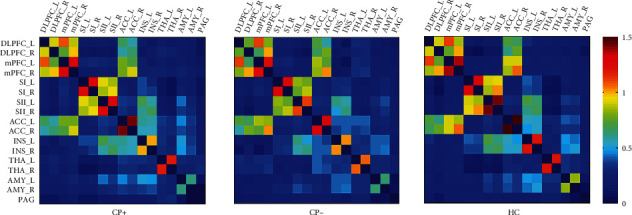
Functional connectivity matrices of the three groups. Colormap shows *z* values of functional connectivity. CP+: lung cancer patients with bone metastases suffering from cancer pain; CP−: lung cancer patients without pain-related complaints; HC: health controls; DLPFC: dorsal lateral prefrontal cortex; mPFC: medial prefrontal cortex; SI: primary somatosensory cortex; SII: secondary somatosensory cortex; ACC: anterior cingulate cortex; INS: insula; THA: thalamus; AMY: amygdala; PAG: periaqueductal gray; R: right; L: left.

**Figure 3:**
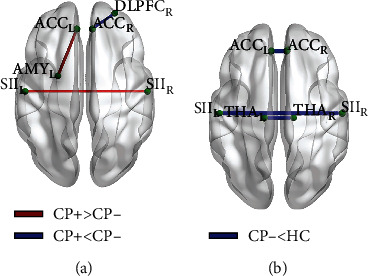
Statistical differences of functional connectivity in intergroup comparisons. (a) Shows the comparison between CP+ and CP−. (b) Shows comparison between CP− and HC. CP+: lung cancer patients with bone metastases suffering from cancer pain; CP−: lung cancer patients without pain-related complaints; HC: health controls; DLPFC: dorsal lateral prefrontal cortex; SII: secondary somatosensory cortex; ACC: anterior cingulate cortex; THA: thalamus; AMY: amygdala; L: left; R: right.

Functional connectivity matrices of the three groups are shown in Figure 2. Statistically differences in functional connectivity are shown in this exploratory study of pain-related brain regions among the three groups (Table 3 and Figure 3). Compared to the CP− group, the CP+ group showed significantly decreased FC between the right DLPFC and the right ACC. Moreover, the CP+ group showed significantly increased FC between the left ACC and the left amygdala, as well as between the bilateral SII. Compared to the HC group, the CP− group showed significantly decreased FC between the bilateral SII, between the bilateral ACC, and between the bilateral thalamus. No significant differences were found between the CP+ and HC groups. Unfortunately, the ANCOVA results of functional connectivity could not bear the multiple comparisons correction (*p* > 0.05/17).

### 3.3. Correlations in the CP+ Group

**Figure 4:**
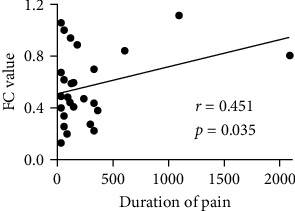
Positive correlation between the functional connectivity of the right DLPFC and the right ACC with the duration of cancer pain in the CP+ group. CP+: lung cancer patients with bone metastases suffering from cancer pain; DLPFC: dorsal lateral prefrontal cortex; ACC: anterior cingulate cortex; FC: functional connectivity.

As shown in Figure 4, in the CP+ group, FC between the right DLPFC and the right ACC was positively correlated with the duration of cancer pain (*r* = 0.451, *p* = 0.035). No significant correlations were found between the FC values and pain intensity.

## 4. Discussion

We report alterations in functional connectivity in pain-related brain regions in lung cancer patients with bone metastasis suffering from cancer pain compared to the control groups and correlations between altered functional connectivity and the duration of cancer pain. The results showed that the altered functional connectivity was mainly concentrated in the DLPFC, ACC, SII, and amygdala in the CP+ group compared to the CP− group. Among these brain regions that showed statistical differences, functional connectivity between the right DLPFC and the right ACC showed a positive correlation with the duration of cancer pain in the CP+ group. In addition, altered functional connectivity was found in the bilateral SII, ACC, and thalamus between the CP− group and the HC group.

We found weaker functional connectivity between the right DLPFC and the right ACC in the CP+ group compared to the CP− group, and this functional connectivity showed a positive correlation with the duration of cancer pain. The DLPFC and ACC are two major cognitive-emotional modulation areas that are known to receive nociceptive input from the periphery and control pain perception by top-down modulation [29]. A previous study demonstrated that after taking analgesics, patients with chronic radicular neuropathic pain were relieved with a reduction in network connectivity between the DLPFC and ACC [11]. Similarly, we found weaker functional connectivity between the right DLPFC and the right ACC in the CP+ group (all CP+ patients had experienced analgesics). With the long duration of cancer pain that had not been cured, this functional connectivity gradually recovered. We speculate that the DLPFC and ACC may be critical brain regions that cause cancer pain to be refractory. Moreover, DLPFC stimulation by repetitive transcranial magnetic stimulation (rTMS) has a potential analgesic effect on chronic pain [30, 31]. This provides a possibility for future studies on the analgesia of cancer pain.

In addition, we found stronger functional connectivity between the left ACC and the left amygdala in the CP+ group compared to the CP− group. The amygdala is a crucial subcortical region thought to contribute to emotional components of pain [32]. Hyperexcitability of the pathway between the basolateral amygdala and the ACC was found in mouse models with neuropathic pain, and inhibiting basolateral amygdala inputs can elicit pain-related aversion [33]. Effective connections were found between the ACC and amygdala in adults during the experience of pain while also experiencing sadness [34]. It is plausible to suggest that stronger functional connectivity between the ACC and amygdala in patients with cancer pain may result from a cerebral response to pain-related aversion.

Moreover, we found stronger functional connectivity between the left SII and the right SII in the CP+ group compared to the CP− group. To our knowledge, SII generally presented stronger functional connectivity with the other pain-related brain regions in patients under pain conditions [12, 35]. Surprisingly, in lung cancer patients with bone metastatic pain, stronger functional connectivity between the SII in both cerebral hemispheres was found. SII is responsible for spatial, tactile, and motor memory associated with sensory experiences [36]. Our finding suggests that somatosensory perception coding between the two hemispheres increased during bone metastatic pain.

In addition, in the CP− group, weaker functional connectivity was found in bilateral SII, ACC, and thalamus compared to the HC group, due to the complexity of lung cancer, brain function susceptible to cancer treatment, psychological factors, and the lung cancer itself [37]. Researchers found that lung cancer patients both with and without chemotherapy had cognitive impairment [38]. Connectivity differences in the default mode network, predominantly in the anterior temporal network and cerebellum network, were found in lung cancer patients versus healthy controls. According to these studies, to eliminate the physiological and psychological effects of lung cancer on brain function in the present study, we used lung cancer patients without pain-related complaints as a control group. Finally, we found no significant differences between the CP+ group and HC group. We supposed that too many confounding factors were present to directly compare the results. The results suggest that the different effects on the brain function may not have simply appeared successively but rather mutually influenced one another, leading to countermeasures. Actually, these results need to be interpreted with caution. Another possible reason is the bias caused by the small sample size.

The limitations of this study should be mentioned. First, our results could not bear the multiple comparisons correction in this exploratory study. However, a previous study found a high false-positive rate when using a stringent threshold for multiple comparison correction and revealed that small *p* values may not yield robust findings [39]. Because of the exploratory nature of this study, multiple comparison correction was not used in the comparisons within the 17 × 17 correlation matrix. Second, we also did not use seed-based global functional connectivity analysis. Instead, we restricted our analysis to a priori selected ROIs. Third, the relatively small sample size of this study may limit its generalizability. However, the sample size here was larger than that reported in previous neuroimaging studies of cancer pain (ranging between 10 and 13 participants) [17, 18]. Last, due to the cross-sectional design of the present study, the direction of causality underlying the observed associations remains unknown. Thus, longitudinal studies will be important in the future.

## 5. Conclusion

These data were the first to show altered brain functional connectivity within pain-related brain regions in lung cancer patients with bone metastatic pain and to reveal its correlation with pain duration. This study may provide a better understanding of bone metastatic pain.

## Figures and Tables

**Table 1:** Templates of the pain-related brain regions.

Templates	Brain region	Abbreviation	Hemisphere
Anatomical Automatic Labeling	Thalamus	THA	L
			R
	Insula	INS	L
			R
	Amygdala	AMY	L
			R
	Anterior cingulate cortex	ACC	L
			R

Juelich Histological Atlas	Primary somatosensory cortex	SI	L
			R
	Secondary somatosensory cortex	SII	L
			R

Brainnetome Atlas	Dorsolateral prefrontal cortex	DLPFC	L
			R
	Medial prefrontal cortex	mPFC	L
			R

Harvard Ascending Arousal Network Atlas	Periaqueductal gray	PAG	/

Abbreviations: MNI: Montreal Neurological Institute; R: right; L: left.

**Table 2:** Demographic and clinical characteristics.

	CP+ (*n* = 27)	CP− (*n* = 27)	HC (*n* = 27)	*p* value
*Demographics*				
Age (years)	60.11 ± 8.45 (45–76)	59.96 ± 7.05 (48–73)	59.00 ± 8.18 (44–74)	0.855
Gender (man : female)	18: 9	18: 9	18: 9	1.000
Education (years)	8.19 ± 3.60 (0–16)	7.59 ± 2.39 (4–12)	11.00 ± 4.24 (0–20)	0.001
Anxiety SAS	36.15 ± 10.06 (25–62)	31.37 ± 4.58 (25–42)	34.15 ± 7.66 (25–47)	0.069
Depression SDS	34.42 ± 10.93 (25–70)	29.70 ± 4.45 (25–43)	33.56 ± 9.40 (25–62)	0.111
*Clinical characteristics*				
Cancer stage, No. (%)				
I	0	3 (11.11%)	NA	NA
II	0	1 (3.70%)	NA	NA
III	0	6 (22.22%)	NA	NA
IV	27 (100%)	17 (62.92%)	NA	NA
Subtype, No. (%)			NA	NA
Adenocarcinoma	16 (59.26%)	15 (55.56%)	NA	NA
Squama cancer	9 (33.33%)	10 (37.04%)	NA	NA
Sarcomatoid carcinoma	0	1 (3.70%)	NA	NA
Small cell cancer	2 (7.41%)	1 (3.70%)	NA	NA
Therapeutic regimen, No. (%)			NA	NA
Chemotherapy	21 (43.75%)	16 (53.33%)	NA	NA
Radiotherapy	10 (20.83%)	4 (13.33%)	NA	NA
Targeted therapy	10 (20.83%)	5 (16.67%)	NA	NA
Surgery	3 (6.25%)	5 (16.67%)	NA	NA
Immunotherapy	1 (2.08%)	0	NA	NA
Chinese medicinal therapy	3 (6.25%)	0	NA	NA
NRS	2.33 ± 1.66 (1–7)	0	0	NA
Duration of cancer pain (days)	265.93 ± 430.36 (20–2095)	NA	NA	NA

Note: values shown are mean ± SD (MIN–MAX) unless noted otherwise. Abbreviation: CP+: lung cancer patients with bone metastases suffering from cancer pain; CP−: lung cancer patients without pain-related complaints; HC: health controls; SD: standard deviation; MIN: minimum; MAX: maximum; BMI: body mass index; SAS: Self-Rating Anxiety Scale; SDS: Self-Rating Depression Scale; NA: not applicable.

**Table 3:** Functional connectivity differences of the pain-related brain regions in three groups.

	ANCOVA		Post hoc test	
Brain region	*F* (*H*) value	*p* value	*t* (*μ*) value	*p* value
DLPFC (R)—ACC (R)	3.297	0.043	−2.400^a^	0.019^a^
ACC (L)—AMY (L)	7.394^∗^	0.025^∗^	2.661^∗^^b^	0.023^∗^^b^
SII (L)—SII (R)	5.769	0.005	2.003^b^	0.049^b^
			−3.377^c^	0.001^c^
ACC (L)—ACC (R)	6.446	0.003	−3.594^c^	0.001^c^
THA (L)—THA (R)	4.493	0.014	−2.980^c^	0.004^c^

∗ means results of nonparametric test; ^a^CP + <CP−; ^b^CP + >CP−; ^c^CP − <HC. Abbreviations: CP+: lung cancer patients with bone metastases suffering from cancer pain; CP−: lung cancer patients without pain-related complaints; HC: health controls; ANCOVA: analysis of covariance; DLPFC: dorsolateral prefrontal cortex; ACC: anterior cingulate gyrus; AMY: amygdala; SII: secondary somatosensory cortex; THA: thalamus; R: right; L: left.

## Data Availability

The study data presented may be made available from the corresponding authors upon reasonable request.
